# Putative Genes Involved in Saikosaponin Biosynthesis in *Bupleurum* Species

**DOI:** 10.3390/ijms140612806

**Published:** 2013-06-19

**Authors:** Tsai-Yun Lin, Chung-Yi Chiou, Shu-Jiau Chiou

**Affiliations:** 1 Institute of Bioinformatics and Structural Biology & Department of Life Science, National Tsing Hua University, No. 101, Sec. 2, Kuang Fu Rd., Hsinchu 30013, Taiwan; E-Mail: d94b42004@ntu.edu.tw; 2 Biomedical Technology and Device Research Laboratories, Industrial Technology Research Institute, No. 321, Sec. 2, Kuang Fu Rd., Hsinchu 30011, Taiwan; E-Mail: ShuJiauChiou@itri.org.tw

**Keywords:** anti-hepatitis, anti-inflammation, *Bupleurum* species, ethylene response factor, medicinal herb, methyl jasmonate (MeJA), saikosaponin, triterpenoid

## Abstract

Alternative medicinal agents, such as the herb *Bupleurum,* are increasingly used in modern medicine to supplement synthetic drugs. First, we present a review of the currently known effects of triterpene saponins-saikosaponins of *Bupleurum* species. The putative biosynthetic pathway of saikosaponins in *Bupleurum* species is summarized, followed by discussions on identification and characterization of genes involved in the biosynthesis of saikosaponins. The purpose is to provide a brief review of gene extraction, functional characterization of isolated genes and assessment of expression patterns of genes encoding enzymes in the process of saikosaponin production in *Bupleurum* species, mainly *B. kaoi*. We focus on the effects of MeJA on saikosaponin production, transcription patterns of genes involved in biosynthesis and on functional depiction.

## 1. Introduction

Alternative medicinal treatments have often been used to supplement more conventional medicines, in part because the herbal medicines are more natural to the human body. Herbs, such as *Bupleurum*, *Ephedra*, *Glycyrrhiza*, *Olea*, *Panax*, *Salvia*, *Scrophularia* and *Scutellaria*, have been used as anti-inflammatories for generations in many countries, especially China. Bark, leaves, flowers, fruits, seeds, stems and roots of herbal plants can be used for therapeutic effects and to improve health. *Bupleurum* roots (Apiaceae family) are commonly used for reducing fever and relieving irritability and are the main ingredient in “minor *Bupleurum* decoction”. Several groups of secondary metabolites isolated from *Bupleurum* species, including triterpenoid saponins (saikosaponins), steroidal saponins, lignans, essential oils and polysaccharides, have been characterized as having relevant biological activity [1]. *Bupleurum* herbs are perennial and have bisexual flowers with five stamens. Their fruits are often cremocarps, and leaves are simple, long, slender and alternate with the entire margin. The *Bupleurum* genus is represented by 180–190 species, which are widely distributed in the Northern hemisphere and commonly used in Eurasia and North Africa for their medicinal properties [2]. Saikosaponin is the most important pharmacological constituent in *Bupleurum* root extract.

Traditional medicines were often produced by multi-component crude drugs, which may have resulted in combinatorial activation and/or suppression effects. Recently, new molecular techniques have been used in plant research, for example, emerging applications of microarray, next generation sequencing and comprehensive analyses of transcriptomes have become widespread [3]. Genes involved in secondary metabolism often work in coordination with other genes at the transcriptional level. A number of gene discovery studies have been successfully conducted using network analysis, especially by integrating gene co-expression network analysis and metabolomic investigation [4]. In addition, next-generation sequencing technologies are currently being utilized in functional genomics investigations of *Arabidopsis* and non-model plant species, including medicinal plants [5,6]. Systems-based approaches are expected to provide important information in medicinal plant research. This review primarily discusses discovery and analysis of the genes involved in saikosaponin biosynthesis from some non-model *Bupleurum* species.

Among the three *Bupleurum* species commonly cultivated in Taiwan, *B. kaoi* Liu, Chao et Chuang is endemic. *Bupleurum falcatum* L. cv. Tainung No.1 is a selected breeding line of *B. falcatum* introduced from Japan, and *B. chinense* DC. is grown predominately in China; its processed herbal materials are imported to Taiwan [7]. A previous report showed that *B. kaoi* exhibited a greater hepatoprotective effect and had larger quantities of saikosaponins a, c and d than *B. falcatum* and *B. chinense* [8]. This article focuses on recent molecular studies of the biological effects, transcript profiling, gene extraction and functional characterization of the above-mentioned *Bupleurum* species.

## 2. Biological Effects of *Bupleurum* Saikosaponins

**Figure 1:**
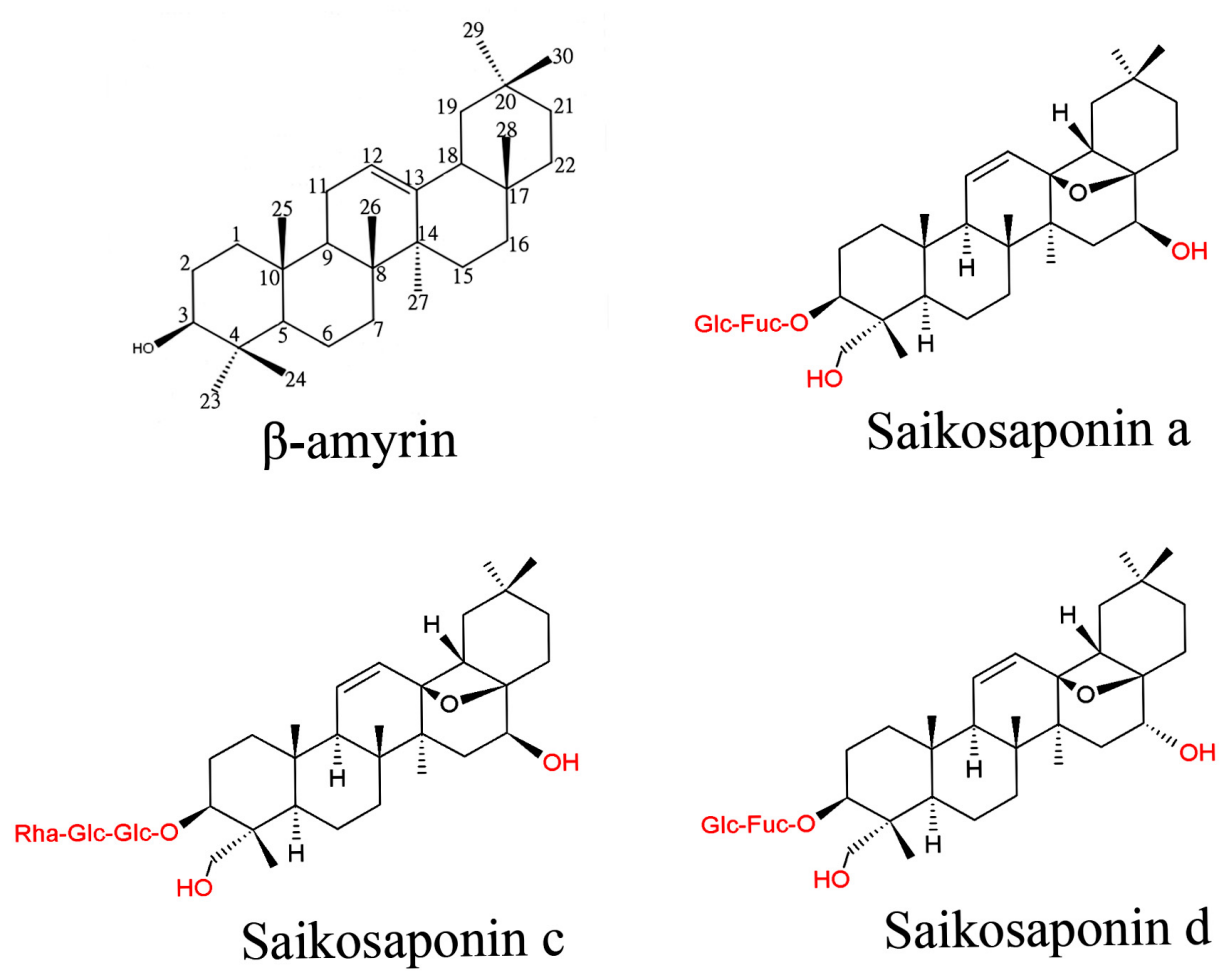
Chemical structures of β-amyrin and saikosaponins a, c and d isolated from *Bupleurum* species.

*Bupleurum* species have been widely used for the treatment of chronic hepatitis and inflammatory diseases. Many saikosaponins display very potent anti-inflammatory, hepatoprotective and immunomodulatory activities both *in vivo* and *in vitro*. Triterpenoid saponins occur widely in plants and exhibit a wide range of structural diversity and biological activity [1]. Many *Bupleurum* plants contain several bioactive components, including saponins, flavonoids, coumarins, fatty acids, steroids, polysaccharides and polyacetylenes. This section focuses on the potential role of the *Bupleurum* triterpenoid saponins (Figure 1), which are synthesized by many different *Bupleurum* species. More than 120 glycosylated oleanane-type and ursane-type saponins have been isolated from the roots of intact plants or from tissue-cultured roots of *Bupleurum* species [9–12]. Oleanane-type saikosaponins a, c and d have been found in nearly all of the *Bupleurum* species tested, with variations in component abundance and total saponin content between different plants and, especially, from different localities [13]. Other saikosaponins with different aglycones or sugar chains have also been isolated from various *Bupleurum* species [14–16].

Numerous studies have demonstrated that most *Bupleurum* extracts or isolated saikosaponins are potentially active compounds and exhibit a wide range of pharmacological actions based on *in vivo* and *in vitro* experimental evidence [17–20]. Root extracts of *B. fruticosum* L. showed hepatoprotective and phagocytosis stimulating effects, and the fraction with promising anti-hepatotoxic activity was identified as saikogenin [17]. *Bupleurum falcatum* has been extensively investigated as an anti-inflammatory drug, and administration of the saikosaponin mixture demonstrated significant activity against acute and chronic inflammation [18]. Inhibitory effects of saikosaponins on d-galactosamine-induced hepatic necrosis were observed in conjunction with a reduction in many hepatic injury markers [19,20]. *Bupleurum chinense* saponins significantly enhanced cellular responses to ovalbumin in mice with low hemolytic effect; thus, saponins could be safely used as an adjuvant with few side effects [21]. Extract of *B. kaoi* showed anti-inflammatory and anti-fibrotic activities followed by anti-oxidant activity, enhanced liver cell regeneration and regulation of interferon-γ and interleukin-10 [8]. Using a 12-*O*-tetradecanoylphorbol-13-acetate (TPA) multiple-dose model for skin chronic inflammation, saponins from *B. rotundifolium* L. were proven to be effective against TPA-induced ear edema in mice [22].

Antiphlogistic activity was found in saikosaponins extracted from *B. rigidum* [23]. Saikosaponin d from the roots of *B. falcatum* increased phagocytosis, lysosomal enzyme activity and stimulated *in vivo* immunological lymphocyte functions [24]. The effect of saikosaponin d on activated mouse T lymphocytes has been studied in relation to nuclear factor (NF)-κB, NF of activated T-cells (NF-AT) and activator protein 1 (AP-1) signaling pathways, cytokine secretion and IL-2 receptor expression. Results from these studies demonstrated that saikosaponin d suppressed activation and proliferation of phorbol myristate acetate (PMA)-stimulated human T-cells *in vitro* [25]. Intravenously administered saikosaponins, obtained from the methanol extract of *B. falcatum*, was found to inhibit the passive cutaneous anaphylaxis reaction in rats and suppressed asthmatic bronchoconstriction in sensitized guinea pigs [26]. Saikosaponin d isolated from *B. falcatum* stimulated the proliferation of MCF-7 cells that were mediated through the estrogen receptor, indicating that saikosaponin may be a type of phytoestrogen [27]. Saikosaponin a inhibited activation and proliferation of T-cells through cell cycle arrest at the G0/G1 phase and induction of apoptosis via the mitochondrial pathway [28]. Production and expression of interleukin-6 and tumor necrosis factor-α in PMA or A23187-stimulated human mast cell (HMC)-1 cells was inhibited by saikosaponin a. Saikosaponin a also suppressed phosphorylation of extracellular signal-regulated kinase, translocation of NF-κB/Rel A into nuclei and degradation of inhibitor of NF-κB (IκB) in cytoplasm. The inactivation of cysteine-aspartic acid protease (caspase)-1 by saikosaponin a in stimulated HMC-1 cells provides a new strategy for the therapeutic treatment of inflammatory diseases [29]. Saikosaponins a and d sensitized cancer cells to cisplatin-induced cytotoxicity through reactive oxygen species-mediated apoptosis, and this chemosensitization can be used as an effective therapeutic strategy [30]. Saikosaponins a, d and e isolated from *B. falcatum* roots exhibited a potent anti-cell adhesive activity on some solid tumor cells [31]. In an *in vitro* study using C6 rat glioma cells, saikosaponin b_2_ (saikogenin d) showed a dual effect: inhibition of Ca^2+^ ionophore A23187-induced PGE2 production and elevation of intracellular free Ca^2+^ concentration [32].

## 3. Putative Biosynthetic Pathway of Saikosaponins in *Bupleurum*

More than 120 oleanane-type and ursane-type saponins have been isolated from *Bupleurum* species [9–17]. Saponin aglycones have closely related oxygenated pentacyclic triterpenoidal structures that can be distinguished only by the positions and numbers of the double bonds in rings C and D and oxygenation patterns in positions 16, 23, 28 and 30 [1]. Triterpenes are synthesized via the mevalonate-dependent isoprenoid pathway in higher plants, and oxidosqualene is a precursor common to the biosynthesis of triterpenoids [33,34]. The first committed step in the synthesis of triterpenoid saponins in plants involves the cyclization of 2,3-oxidosqualene by oxidosqualene cyclases (OSC). In higher plants, cyclization of oxidosqualene to β-amyrin is catalyzed by β-amyrin synthase (BAS) and to cycloartenol by cycloartenol synthase (CAS) [35,36]. The *Bupleurum* BAS is presumed to be the enzyme that catalyzes the first committed step in saikosaponin biosynthesis, similar to that in *Medicago truncatula* Gaertn. [36,37].

After triterpene backbone biosynthesis, the downstream processes include a set of cytochrome P450-dependent (P450) hydroxylations/oxidations and several glycosyl transfer reactions catalyzed by glycosyltransferases (GT) [33]. Enzymatic activity of UDP-glucuronosyltransferase was detected in excised root cultures of *Gypsophila paniculata* L. that displayed a positive correlation with the amount of triterpenoid saponins [38]. It has been proposed that the oxidation/hydroxylation catalyzed by specific cytochrome P450s and glycosyl transfer by uridine diphosphate (UDP)-glycosyltransferases (UGTs) are involved in the reactions downstream of the saikosaponin biosynthetic pathway after the cyclization of β-amyrin [35,39]. The fact that plant P450s and UGTs are encoded by a large number of multigene families causes difficulties in predicting the potential involvement of specific P450s and UGTs in saponin biosynthesis. Hundreds of putative genes coding for P450 have been assigned to *Lotus japonicus* L. [40], *M. truncatula* [41], *A. thaliana* and rice genomes [42]. *Glycyrrhiza glabra* L. CYP88D6 has been characterized as a cytochrome P450 monooxygenase using *in vitro* enzymatic activity assays. The *G. glabra* CYP88D6 is a β-amyrin oxidase that catalyzes the sequential two-step oxidation of β-amyrin at C11 to produce 11-oxo-β-amyrin in the glycyrrhizin pathway [43]. CYP88D6 expression was detected in roots and stolons, but not in leaves or stems, which is consistent with the accumulation pattern of glycyrrhizin in plants. A second relevant P450 (CYP72A154) was identified as responsible for oxidation at the C30, C22 and C29 positions in glycyrrhizin biosynthesis [44]. The soybean β-amyrin 24-hydroxylase was also functionally characterized [45]. In addition, *M. truncatula* CYP72A63, which has high sequence similarity with *Glycyrrhiza* CYP72A154, is able to catalyze C30 oxidation of β-amyrin by *in vivo* assay using yeast cells [44].

The *M. truncatula* UGT73K1 and UGT71G1 have been found to be involved in terpenoid biosynthesis [46]. Biosynthesis of many classes of secondary plant metabolites are induced by the plant hormones, methyl jasmonate (MeJA) and jasmonic acid (JA) [47], and MeJA markedly promoted saikosaponin production [48]. MeJA regulates the accumulation of phenolics, terpenes and alkaloids in cell suspension cultures of many plant species and initiates *de novo* transcription of genes encoding phenylalanine ammonia lyase [49]. Transcripts encoding BAS, a group of cytochrome P450 hydroxylases, and several UGTs were increased by MeJA application in adventitious root cultures of *B. kaoi*, which is correlated with increased saikosaponin production [50]. Some *B. chinense* P450s and UGTs exhibit tissue-specific response to MeJA, and a putative saikosaponin biosynthetic pathway was derived from studies in *B. chinense* [51], *B. falcatum* [52] and *B. kaoi* [50]. Among 150 CYP450 and 235 GT sequences found in *Panax quinquefolius* L., one CYP450 and four UGTs are putative candidates involved in ginsenoside biosynthesis through MeJA induction and tissue-specific expression pattern analysis [53].

## 4. Identification and Characterization of Genes Involved in *Bupleurum* Saikosaponin Biosynthesis

**Figure 2:**
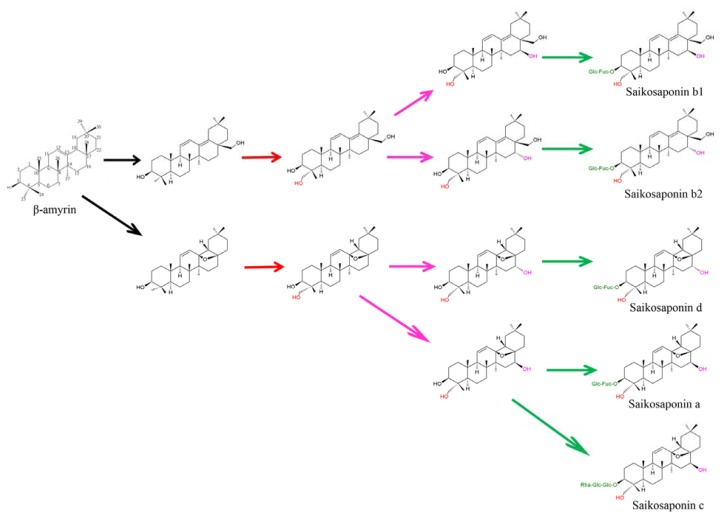
Putative saikosaponin biosynthetic pathway in *B. kaoi*. The tentative steps are proposed with chemical structures of biosynthetic intermediates between β-amyrin and saikosaponins. β-amyrin undergoes various modifications, including P450-catalyzed oxidation and uridine diphosphate (UDP)-glycosyltransferase (UGT)-catalyzed glycosylation. Black arrows represent undefined oxidation at C11/C12 and C13/C18 in saikosaponin b and epoxidation at C13/C28 in saikosaponin a/c/d. Red arrows indicate the reactions possibly catalyzed by C23 hydroxylases. Pink arrows indicate the reactions catalyzed by C16 hydroxylase. Green arrows indicate the sequential glycosylation at the C3 position.

*Bupleurum kaoi* has 12 pairs of chromosomes and a genome size of about 7.3 × 10^8^ bp per copy, while *B. chinense* has a size of about 1.1 × 10^9^ bp per copy. Overexpression of important enzymes in the triterpenoid biosynthetic pathway is a tactic to control saikosaponin production, and strategies can be applied through upregulation of target genes encoding enzymes involved in triterpenoid biosynthesis. Although geranylgeranyl pyrophosphate may be used for triterpenoids generation, the key steps in the saikosaponin biosynthesis in *B. kaoi* are likely the cyclization of 2,3-oxidosqulene catalyzed by BAS and further modification by P450 and GTs [35,50]. Exogenous application of MeJA resulted in a two-fold increase in the *BkBAS* mRNA level and markedly enhanced the level of *BkUGT* mRNA (98-fold), which has very likely contributed to an increase of saikosaponin [50]. In the major bioactive constituents of *Bupleurum* species, the structure of saikosaponins a/d/c is a 13,28-epoxy-16,23-dihydroxyolean with a C11/C12 double bond, and the saikosaponin b structure is a 16,23,28-trihydroxyolean with C11/C12 and C13/C18 double bonds. Saikosaponins a has a 16β-hydroxyl group, which is different from the 16α-hydroxyl group in saikosaponin d. Similarly, saikosaponin b_1_ differed from b_2_ by having a 16β-hydroxyl group. To the best of our knowledge, we present here putative pathways to saikosaponins from β-amyrin in *B. kaoi* (Figure 2). For production of saikosaponins, P450 enzymes (β-amyrin oxidases) are necessary to catalyze oxidation of β-amyrin to form 13,28-epoxy and the C11/C12 double bond structure or to form two double bonds at C11/C12 and C13/C18. Hydroxylation at C16, C23 and/or other positions may be catalyzed by different P450 enzymes, and glycosylation at C3 is catalyzed by UGT. The following parts of this section will summarize our current knowledge regarding the above-mentioned enzymes.

### 4.1. The Oxidosqualene Cyclase (OSC) Genes

A number of oxidosqualene cyclase (OSC) genes from model plants, crops and medicinal plants have been cloned and functionally characterized [36,54–58]. These OSCs are separated into three distinct groups; BAS proteins, CAS proteins and other OSC enzymes. The *B. kaoi BkBAS* gene encodes an OSC enzyme that cyclizes oxidosqualene to β-amyrin. The deduced BkBAS peptide sequence was subjected to a homology search in The Arabidopsis Information Source (TAIR) using the WU-BLAST tool, and 14 putative OSCs were obtained with an E-value cutoff of 10^−220^. The BkBAS peptide shares 75% identity with *Arabidopsis* AtBAS (AT1G78950), which has been identified as a product-specific BAS by heterologous expression in *Saccharomyces cerevisiae* [59]. The BcBAS and BfBAS peptides retrieved from NCBI are partial sequences and share 78 and 75% identity with BkBAS in the corresponding regions. The *B. kaoi* BkBAS protein shares high sequence identity with ginseng PgBAS (83%) [60], tomato SlBAS (82%) [61] and poplar PtBAS (82%). Moreover, *BAS* genes from *Glycyrrhiza echinata* L. [60], *Pisum sativum* L. [62], *Saponaria vaccaria* L. [63] and *Avena sativa* L. [64,65] have been functionally characterized. Among the *Arabidopsis* OSC genes, *ATBAS* (AT1G78950), *ATCAMS1* (AT1G78955, camelliol C synthase 1), *ATLUP2* (AT1G78960, lupeol synthase 2) and *ATLUP1* (AT1G78970, lupeol synthase 1) genes are clustered on chromosome 1. At1g78970 was found to encode a multifunctional OSC yielding more than nine products, including β-amyrin, α-amyrin and lupeol [66], and At1g78955 encodes CAMS1, a camelliol C synthase [67].

**Figure 3:**
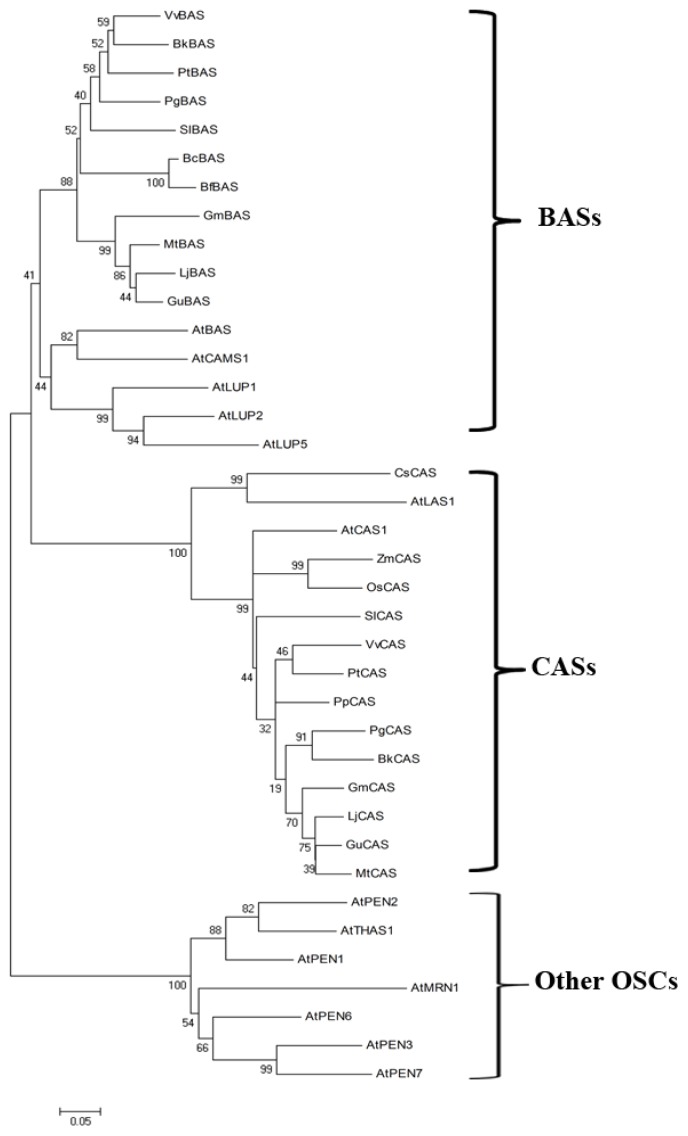
The phylogenetic tree of oxidosqualene cyclase (OSC) proteins was generated using Clustal W with 1,000 bootstraps based on the alignment in the MEGA5.0 software using the deduced amino acid sequence of BkOSC and a set of OSC proteins. Bootstrap values are shown at nodes. The scale bar denotes 0.05 nucleotide substitutions per site. Sequence data of the analyzed OSC proteins in this article can be found in the GenBank database under the following gene names and accession numbers: *A. thaliana AtBAS* (NM_106544, AT1G78950), *AtCAS1* (NM_126681, AT2G07050), *AtPEN2* (NP_193272, AT4G15370), *AtPEN1* (NM_117622, AT4G15340), *AtTHAS1* (NP_001078733, AT5G48010), *AtPEN6* (NP_177971, AT1G78500), *AtPEN3* (NP_198464, AT5G36150), *AtMRN1* (NP_199074, AT5G42600), *AtPEN7* (NP_001154653, AT3G29255), *AtCAMS1* (NP_683508, AT1G78955), *AtLUP2* (NP_178017, AT1G78960), *AtLUP1* (NP_178018, AT1G78970), *AtLUP5* (NP_176868, AT1G66960), *AtLAS1* (NP_190099, AT3G45130); *B. chinense BcBAS* (ABY90140); *B. kaoi BkBAS* (unpublished data); *B. falcatum*, *BfBAS* (ABA01561); *Cucumis sativus CsCAS* (XP_004137362); *Glycine max GmBAS* (XP_003521100) and *GmCAS* (XP_003516849); *Glycyrrhiza uralensis GuBAS* (ACV21067) and *G. glabra GuCAS* (Q9SXV6); *L. japonicas LjBAS* (BAE53429) and *LjCAS* (BAE53431); *M. truncatula MtBAS* (AES86318) and *MtCAS* (XP_003610947); *Oryza sativa OsCAS* (AAF03375); *P. ginseng PgBAS* (O82140) and *PgCAS* (O82139); *Populus trichocarpa PtBAS* (XP_002330453) and *PtCAS* (XP_002308131); *Prunus persica PpCAS* (EMJ26439); *Solanum lycopersicum SlBAS* (NP_001234604) and *SlCAS* (NP_001233784); *Vitis vinifera VvBAS* (XP_002270934) and *VvCAS* (XP_002264372); *Zea mays ZmCAS* (AFW66542).

*Avena sativa* oxidosqualene cyclase AsBAS1 catalyzes the first committed step in the synthesis of antifungal triterpenoid saponins that accumulate in oat roots [65]. Orthologs of AsBAS1 are absent from modern cereals; thus, this gene could be utilized to enhance disease resistance in crop plants. Tomato SlTTS1 (SlBAS) forms β-amyrin as its sole product, while SlTTS2 catalyzes the formation of seven different triterpenoids, with δ-amyrin as the major product [61], and overexpression of SlTTS1 and SlTTS2 led to increased accumulation of cuticular triterpenoids. The NCBI-BLAST search using BkBAS and BkCAS peptides as the query generated a large number of OSC proteins with high sequence similarity; thus, only the OSC peptides from *A. thaliana* and the BAS and CAS peptides from *B. chinense*, *B. falcatum*, *B. kaoi*, some model plants and herbal plants were used in phylogenetic analysis (Figure 3). Some BASs have been described as monofunctional enzymes [60,68], while some multifunctional OSCs, such as ATLUP2, also catalyze the synthesis of δ-amyrin, α-amyrin or lupeol (ATLUP2) [61,69].

### 4.2. The Cytochrome P450 Monooxygenases/Hydroxylases Genes

Cytochrome P450 monooxygenases/hydroxylases (P450s), which are enzymes found in plants, human and bacteria, catalyze the oxidation of various substrates by oxygen and NAD(P)H [42,70]. Plant P450s catalyze a wide variety of monooxygenation/hydroxylation reactions in secondary metabolism [45,71]. Soybean CYP93E1 was identified as β-amyrin C-24 hydroxylase, which is involved in soyasaponin biosynthesis [46]. Glycyrrhizin accumulated in roots and stolons of *Glycyrrhiza* species exhibits a wide range of pharmacological activities. The β-amyrin oxidase, CYP88D6, was characterized in both *G. glabra* and *G. uralensis. Glycyrrhiza uralensis* CYP93E3 was identified as β-amyrin 24-hydroxylase, and CYP72A154 was found to catalyze C30 oxidation of β-amyrin [44]. *Medicago truncatula* CYP716A12 was characterized as a multifunctional enzyme with β-amyrin 28-oxidase, α-amyrin 28-oxidase and lupeol 28-oxidase activities. Homologs of CYP716A12 were also identified in grape (CYP716A15 and CYP716A17), indicating that the function of the CYP716A subfamily among plants is highly conserved [72]. The *Arabidopsis AtBR6ox* and tomato *Dwarf* gene were expressed in yeast, and both were verified as encoding steroid-6-oxidases with a broad substrate specificity [73]. The *Arabidopsis DWF4* gene coding for CYP90B1 was expressed in *Escherichia coli* and was confirmed to function as a steroid C-22 hydroxylase enzyme [74,75]. Both *Arabidopsis* CYP90C1 and CYP90D1 catalyze C-23 hydroxylation of various 22-hydroxylated BRs [76]. It is expected that *B. kaoi* contains an enzyme similar to β-amyrin oxidase for catalyzing the oxidation of β-amyrin to produce 13β,28-epoxy-oleanane aglycone. Then, sequential hydroxylation may be catalyzed by a set of different P450 enzymes, and glycosylation at C3 is catalyzed by various UGTs (Figure 2). The NCBI-BLAST search using the BkCYP clone as the query suggests that this gene may encode a CYP712A1 peptide. The predicted peptide of BkCYP shares 99% sequence identity with the *B. chinense* cytochrome, P450 CYP712F1, and 38% identity with a predicted β-amyrin 24-hydroxylase (XP_002532402) in *V. vinifera* and may function as a 23-hydroxylase.

### 4.3. The UDP-Glycosyltransferase Genes

Glycosylation contributes to the highly diverse nature of plant secondary metabolites. UGTs catalyze the transfer of sugar moieties to a wide range of acceptor molecules of terpenoids and steroids *in planta* [77,78]. As a key enzyme involved in the modification of plant secondary metabolites, glycosylation produces a huge diversity of low molecular weight natural products [79]. Studies combining mutagenesis, molecular modeling and structural analysis may identify the residues and domain structures that are responsible for the enzymatic activity and substrate specificity of plant UGTs. Here, we describe the functional characterization of some plant UGTs that are thought to be involved in saikosaponin production.

A total of 141 loci matches with 167 distinct gene models were found using the term “glycosyltransferase” to search the database in TAIR, indicating that the *Arabidopsis* genome has more than a hundred UGT members. We previously isolated three *B. kaoi* cDNAs (unpublished data) putatively encoding BkUGT85A proteins that share high amino acid sequence identity with *Arabidopsis* AtUGT85A1 (AT1G22400, 51.5%), AtUGT85A2 (AT1G22360, 50%) and AtUGT85A5 (AT1G22370, 59%) based on similarity search with an E-value cutoff of 10^−101^. These putative UGT85A proteins contain a plant secondary product glycosyltransferase (PSPG) motif that has been shown to be involved in sugar donor binding of UGTs and is highly conserved in the UGT family [80].

A comprehensive gene expression clustering analysis was used to identify candidate genes involved in the hydroxylation and glycosylation of the triterpene skeleton in the model legume *M. truncatula* [46,55]. Expression of *M. truncatula* UGT73F3 in *E. coli* showed its specificity for multiple sapogenins, and UGT73F3 was confirmed to glucosylate hederagenin at the C28 position [55]. Soybean group A saponins are subdivided by a difference in the C-22 sugar chains [81]. Saponin Aa has a 2,3,4-tri-*O*-acetyl-β-d-xylopyranosyl(1→3)-α-l-arabinopyranosyl sugar chain, and saponin Ab has 2,3,4,6-tetra-*O*-acetyl-β-d-glucopyranosyl(1→3)-α-l-arabinopyranosyl sugar chain. Codominant alleles at *Sg-1* locus, *Sg-1a* and *Sg-1b* control accumulation of saponins Aa and Ab, respectively [81,82]. The *Sg-1a* allele encodes the xylosyltransferase, UGT73F4, whereas *Sg-1b* encodes the glucosyltransferase, UGT73F2, and Ser-138 in Sg-1a and Gly-138 in Sg-1b proteins are crucial residues for their respective sugar donor specificities [82]. *Medicago truncatula* UGT73K1 has specificity for hederagenin and soyasapogenols B and E, and UGT71G1 has specificity for medicagenic acid [46]. Through the analysis of the transcriptome of *P. notoginseng* root, candidate cytochrome P450 and UDP-glycosyltransferase genes involved in hydroxylation or glycosylation of aglycones for triterpene saponin biosynthesis were discovered [83].

*Solanum aculeatissimum SaGT4A* encodes a functional UGT that catalyzes the glucosylation of both spirostane-type steroidal saponins and spirosolane-type steroidal alkaloids [84]. *Solanum tuberosum* UGT StSGT prefers UDP-galactose to UDP-glucose as a sugar donor, whereas SaGT4A exclusively exhibited glucosyltransferase activity [85]. Differences in SaGT4A and StSGT amino acid sequences (75% homology) do not simply reflect their distinct sugar donor specificities, and slight variation in the amino acid sequence may lead to distortion of the catalytic site of the enzyme, resulting in the loss of function [85]. It is hypothesized that glycosylation of oleanane aglycone at C3 may be catalyzed by the putative BkUGT85As, which will be studied by heterologous expression in *E. coli*. One of the deduced BkUGT85A peptides shares 90% identity with that of the *B. chinense* UGT (AFM52193), and the other shares 67% identity with the *Stevia rebaudiana* UGT85A8 (AAR06913). The huge biodiversity of saponins can be explored effectively through understanding the enzymes involved in each specific step of the synthesis or modification of the triterpene or steroid aglycone backbone.

### 4.4. Genes Involved in Reactions Upstream of the First Committed Step

**Figure 4:**
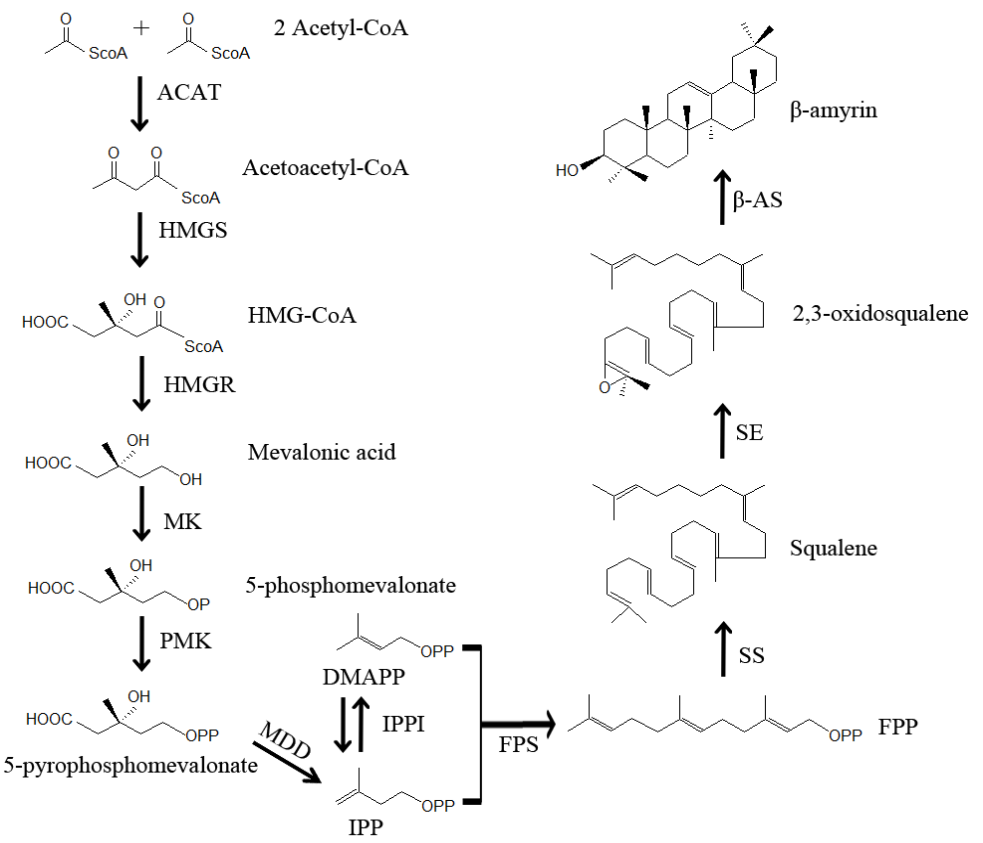
Early steps in saikosaponin biosynthetic pathway. ACAT, acetyl-CoA acetyltransferase; β-AS, β-amyrin synthase; DMAPP, dimethylallyl pyrophosphate; FPS, farnesyl diphosphate synthase; FPP, farnesyl pyrophosphate; HMG-CoA, 3-hydroxy-3-methylglutaryl-CoA; HMGR, HMG-CoA reductase; HMGS, HMG-CoA synthase; IPP, isopentenyl pyrophosphate; IPPI, isopentenyl diphosphate isomerase; MDD, mevalonate diphosphate decarboxylase; MK, mevalonate kinase; PMK, phosphomevalonate kinase; SE, squalene epoxidase; SS, squalene synthase.

The first committed step in the biosynthesis of triterpenoid saponins is the cyclization of 2,3-oxidosqualene, a common intermediate in the biosynthesis of terpenoids that marks a branching point for both triterpenoid and phytosterols biosynthesis [60,86]. In the past, saikosaponin biosynthesis in plants had been investigated at the molecular level, and its biosynthetic pathway has been established [50–52,87]. As illustrated in Figure 4, the first compound for triterpenoids biosynthesis is formed through the condensation of two molecules of acetyl-CoA into acetoacetyl-CoA, catalyzed by acetyl-CoA acetyltransferase (ACAT). Acetoacetyl-CoA condenses with acetyl-CoA to form 3-hydroxy-3-methylglutaryl-CoA (HMG-CoA) by HMG-CoA synthase (HMGS). Reduction of HMG-CoA into mevalonic acid is catalyzed by HMG-CoA reductase (HMGR). The phosphorylation of mevalonic acid is catalyzed by mevalonate kinase (MK) to form 5-phosphomevalonate, and 5-phosphomevalonate is pyrophosphorylated by phosphomevalonate kinase (PMK) to yield 5-pyrophosphomevalonate. The decarboxylation of 5-pyrophosphomevalonate is catalyzed by mevalonate diphosphate decarboxylase (MDD) to form isopentenyl pyrophosphate (IPP). Isomerization of IPP is carried out by IPP isomerase (IPPI) to form dimethylallyl pyrophosphate (DMAPP), and two IPP molecules condense with DMAPP to form farnesyl pyrophosphate (FPP) catalyzed by farnesyl diphosphate synthase (FPS). Then, two molecules of FPP are converted into squalene by squalene synthase (SS). An epoxide group is introduced into squalene by squalene epoxidase (SE) to form 2,3-oxidosqualene, and BAS converts 2,3-oxidosqualene to β-amyrin. Almost all β-amyrins undergo several modifications by UGT and P450 to form saikosaponins.

## 5. Unique and Overlapping Expression Patterns of Genes Involved in Saikosaponin Biosynthesis

Saikosaponin biosynthesis may involve three key enzymes: BAS, which constructs the basic triterpenoid skeletons; cytochrome P450s, which mediate oxidations; and UGTs, which catalyze glycosylations. The *Arabidopsis OSC* gene encoding thalianol synthase 1 (*AT5G48010*), is flanked by *CYP450* genes encoding thalianol hydroxylase (*At5g48000*) and thalian-diol desaturase (*At5g47990*); and these genes are contiguously coexpressed [77]. This study raises the question of whether the expression patterns of genes encoding biosynthetic enzymes required for consecutive steps in the synthesis and modification of saikosaponin are highly correlated. It is important to know whether the genes involved in saikosaponin biosynthesis show spatially or temporally differential expression.

**Figure 5:**
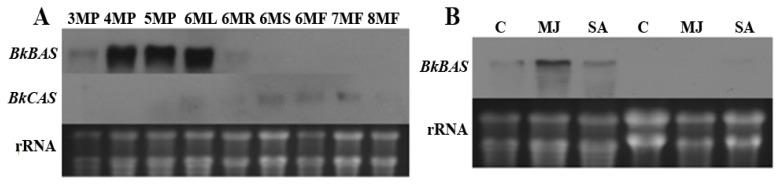
Northern blot analyses of transcript levels of *BkBAS* and *BkCAS* genes in various tissues of *B. kaoi* at different developmental stages (**A**) and in three-month-old plants or adventitious roots responded to 100 μM MeJA or SA (**B**). Total RNA (20 μg) was separated on a 1.2% agarose-formaldehyde gel, blotted onto nylon membrane and hybridized to DIG-labeled *BkBAS* and *BkCAS* probe. M, months; P, whole plant; R, root; S, stem; L, leaf; F, flower; 18S rRNA gene was used for the standard of RNA loading.

Before three months of age, *BkBAS* transcripts are undetectable or barely detectable in *B. kaoi* plants with Northern blotting. Accumulation of *BkBAS* transcripts was found in plants older than three months, primarily in leaves, a smaller amount in roots and below the detection level in stems and flowers (Figure 5A). Levels of *BkCAS* transcripts were low during all examined developmental stages. Northern blot analysis showed that the level of *BkBAS* mRNA in three-month-old plants can be enhanced by exogenous application of 100 μM MeJA for 24 h, but not by salicylic acid (SA) (Figure 5B). However, *BkBAS* transcripts in adventitious roots remained at undetectable or barely detectable levels after MeJA or SA treatment (Figure 5B). Functional characterizations of *S. vaccaria SvBAS* and *UGT74M1* genes were performed by expression in yeast and *E. coli*, respectively. The *S. vaccaria SvBAS* gene is also predominantly expressed in leaves, whereas *UGT74M1* is expressed in roots and leaves [63]. Expression of licorice *CYP88D6* was only detected in roots and stolons by RT-PCR, which is consistent with the accumulation pattern of glycyrrhizin in plants [43]. *Pisum sativum* PsUGT1 expression in meristematic cells is induced in conjunction with mitosis and is required for normal development of transgenic pea hairy roots and alfalfa plants [88]. A total of 137 UGT genes were identified from flax (*Linum usitatissimum* L.) using the conserved PSPG box, and the encoded proteins were clustered into 14 major groups. 100 genes were expressed in 15 tissues with varied expression profiles [88].

The mRNA of a squalene synthase (BfSS1) is ubiquitously expressed in *B. falcatum* plants and markedly increased in roots after MeJA treatment. Enhanced production of both phytosterol and saikosaponins in roots was verified via transgenic plants overexpressing the *BfSS1* gene. Overexpressing the *BfSS1* gene increased mRNA accumulation of squalene epoxidase and cycloartenol synthase, but decreased the level of *BAS* mRNA [52]. A red beet (*Beta vulgaris* L.) UGT gene, *BvGT*, was found to be induced by wounding, bacterial infiltration and oxidative stress. *BvGT* has a high similarity to *Dorotheanthus bellidiformis* betanidin-5 GT involved in betacyanin synthesis, and transient expression of a *BvGT* antisense construct caused the reduction of *BvGT* transcript accumulation and betanin synthesis [89]. Understanding both the expression patterns and the elicitor responses of genes involved in saikosaponin biosynthesis opens the door to uncovering regulatory mechanisms and to increasing target products.

## 6. Functional Characterization of ERF Genes Extracted from *B. kaoi*

Plant secondary metabolites are synthesized in spatial- and temporal-specific patterns and in response to various environmental stimuli. Transcription factors involved in secondary metabolism include the MYB, bHLH, AP2/ERF, WRKY and SPL (SQUAMOSA promoter binding protein-like) families [6]. For example, *Catharanthus roseus* (L.) G. Don produces terpenoid indole alkaloids (TIAs). Overexpression of *CrWRKY1* in *C. roseus* hairy roots upregulated several key TIA pathway genes and the transcriptional repressors, ZCT1, ZCT2 and ZCT3, whereas the activators ORCA2, ORCA3 and CrMYC2 were repressed. The preferential expression of CrWRKY1 in roots upregulates TIA biosynthesis in *C. roseus* plants [90]. The DNA-binding-with-one-finger (DOF) transcription factor OBP2 is expressed in *Arabidopsis* organs, and its expression is responsive to herbivores, pathogens and JA. Overexpression of OBP2 activates expression of CYP83B1, showing that OBP2 is part of a regulatory network controlling glucosinolate biosynthesis in *Arabidopsis* [91]. The *Arabidopsis* MYB transcription factor, ATR1, controls the enzymes that synthesize production of glucosinolates, including CYP79B2, CYP79B3 and CYP83B1, and the accumulation of indolyl glucosinolates [92].

Effective protection against necrotrophic pathogens is often regulated by defense responses activated by JA and ethylene signaling. The 147 members of the *Arabidopsis* AP2/ERF superfamily [93,94] have been divided into four families: ethylene response factor (ERF), dehydration-responsive element-binding protein (DREB), apetala (AP2), related to ABI3/VP1 (RAV) and a solo gene, At4g13040. The ERF genes isolated from *B. kaoi* shares high similarity with the *Arabidopsis* ERF genes, and their deduced peptides all contain a single AP2/ERF domain [94,95]. The AP2/ERF domain is involved in diverse functions in cellular processes, including hormonal signal transduction [96] and responds to biotic [97] and abiotic stress [93,98]. ERF proteins may integrate signals from JA and ethylene pathways in plant disease resistance [99,100]. Overexpression of ERF proteins in *Arabidopsis* plants led to increased expression of PDF1.2, ChiB and Thi2.1 and increased resistance to both necrotrophic [99,101] and biotrophic pathogens [100]. Enhancement of disease resistance in plants has been achieved by overexpressing ERF proteins, such as *Arabidopsis* AtERF1 [101,102], AtERF2 [100] and B *kaoi* BkERF1/2.2 [95]. The rapid activation of *BkERF* transcription after MeJA treatment may lead to the expression of a subset of defense genes [95].

Our previous studies demonstrated that *B. kaoi* ERFs activate defense genes and that the increased gene expression correlates with increased resistance to *Botrytis cinerea* [95]. The expression of ERF genes in both homologous and heterologous transgenic systems suggests that these genes might be generally useful in conferring increased disease resistance to diverse plant species. *Arabidopsis thaliana* ERF6 is a substrate for MPK3/MPK6, which plays a critical role in plant disease resistance by regulating multiple defense responses and resistance to the necrotrophic fungal pathogen, *B. cinerea* [103]. It is possible that other ERFs are subjected to phosphorylation in the same way as *Arabidopsis* ERF6. Expression of *BkBAS*, *BkUGT85A* and *BkCYP* was only found in root tissue, and the saikosaponins mainly accumulate in root. Our previous study showed that MeJA can induce saikosaponin contents in adventitious roots. This raised the question whether the above-mentioned genes can be induced by MeJA. Significant increase of *BkUGT85A* transcript level was observed 2 h after application of 0.1 mM MeJA, and *BkPCYP* transcripts increased 4 h after treatment. The increase of both *BkUGT85A* and *BkCYP* mRNA levels by MeJA treatment remained up to 24 h (unpublished data). Similar events were observed in two JA-responsive AP2 family transcription factors, AaERF1 and AaERF2, from *Artemisia annua* L, which are able to bind to specific motifs present in the promoters of amorpha-4,11-diene synthase (ADS, a sesquiterpene synthase) and CYP71AV1 (a P450 monooxygenase). ADS and CYP71AV1 are two key enzymes in the artemisinin biosynthetic pathway. Transgenic *A. annua* plants overexpressing either transcription factor showed elevated transcript levels of both *ADS* and *CYP71AV1* and increased accumulation of artemisinin and artemisinic acid [104]. The level of *AtP450* mRNA increased in the transgenic *Arabidopsis* plants overexpressing either BkERF, suggesting that ERFs might be involved in saikosaponin biosynthesis.

## 7. Conclusions

The studies discussed here illustrate the putative biosynthetic pathways of bioactive compounds in *Bupleurum* species and show evidence of the effects of *Bupleurum* components. Stable *B. kaoi* cell cultures in combination with MeJA treatment have significantly increased the production of saikosaponins and transcripts of a set of genes coding for enzymes involved in saikosaponins biosynthesis and some transcription factors. BkERFs transcription factors activate defense genes, and the increased gene expression correlates with increased plant defense responses in both homologous and heterologous transgenic systems. Transcript profiling coupled with characterization of gene function can provide insight into the molecular mechanisms underlying plant secondary metabolism. Integrated analysis of the transcriptome and metabolome is powerful, but not sufficient for the molecular characterization of saponin biosynthesis in *Bupleurum* species. To apply RNA sequencing and gene mining systemically requires extensive sequencing of mRNA and protein products. Only a subset of all genes in a genome is expressed at any given time; thus, it is difficult to collect all of the genes in an organism. The high-throughput experimental data may contain small sample sizes, high noise levels and outliers. It is likely that the ensemble databases are incomplete and contain significant amounts of erroneous data. In the future, it will be possible to apply homologous or heterologous transgenic systems to introduce the regulatory genes controlling the biosynthesis of bioactive components into plants. A better understanding of the regulation of gene expression and modification in the biosynthetic pathways is necessary for successful gene manipulation. Employing a combination of metabolomics, transcriptomics and proteomics will help in exploring the regulatory systems found in these medicinal plants and are of great value in the production and use of saikosaponins in medicine.
